# Genetic characterization of thymoma

**DOI:** 10.1038/s41598-019-38878-z

**Published:** 2019-02-20

**Authors:** Lei Yu, Ji Ke, Xin Du, Zhen Yu, Di Gao

**Affiliations:** Department of Thoracic Surgery, Beijing Tongren Hospital, Capital Medical University, Beijing, China

## Abstract

Thymoma represents the most common anterior mediastinal compartment neoplasm, originating from the epithelial cell population in the thymus. Various histological types of thymoma feature different clinical characteristics. Furthermore, thymoma is frequently associated with autoimmune disorders, esp. myasthenia gravis (MG). However, the underlying molecular tumourigenesis of thymoma remains largely unknown. The goal of our current study is to demonstrate the underlying genetic abberations in thymoma, so as to understand the possible cause of MG in thymoma patients. By using CapitalBio mRNA microarray analysis, we analyzed 31 cases of thymoma including 5 cases of type AB thymoma, 6 B1-type cases, 12 B2-type cases, 5 B2B3-type cases and 3 type-B3 cases. 6 cases of thymoma were not associated with myasthenia gravis, while 25 cases were with myasthenia gravis. By comparisons between thymoma and the paratumoral tissues, differentially expressed genes were identified preliminarily. Among them, 292 genes increased more than 2-fold, 2 genes more than 5-fold. On the other hand, 596 genes were decreased more than 2-fold, 6 genes more than 20-fold. Interestingly, among these genes upregulated more than 2-fold, 6 driver genes (FANCI, NCAPD3, NCAPG, OXCT1, EPHA1 and MCM2) were formerly reported as driver oncogenes. This microarray results were further confirmed through real-time PCR. 8 most dysregulated genes were verified: E2F2, EPHA1, CCL25 and MCM2 were upregulated; and IL6, FABP4, CD36 and MYOC were downregulated. Supervised clustering heat map analysis of 2-fold upregulated and 2-fold downregulated genes revealed 6 distinct clusters. Strikingly, we found that cluster 1 was composed of two type-B2 thymoma; and cluster 6 was three type-B2/B3 thymoma. KEGG database analysis revealed possible genetic mechanisms of thymoma and functional process. We further compared gene expression pattern between thymoma with and without MG, and found 5 genes were upregulated more than 2-fold, more than 30 genes were downregulated more than 2-fold. KEGG analysis revealed 2 important signaling pathways with more than 2-fold upregulated genes (TGF- beta signaling pathway and HTLV-I signaling pathway) as differially functioning between MG positive and negative thymomas. Real-time PCR analysis confirmed that CCL25 was upregulated; and MYC, GADD45B, TNFRSF12 downregulated in thymoma with MG. Our study thus provided important genetic information on thymoma. It shed light on the molecular bases for analyzing the functional process of thymoma and finding potential biomarkers for pathological categorizing and treatment. Our work may provide important clues in understanding possible causes of MG in thymoma patients.

## Introduction

Thymoma represents the most common anterior mediastinal neoplasm, originating from the epithelial cell population in the thymus. Thymoma is characterized with unique features in comparison to other epithelially originated tumors. First of all, it has been generally considered to have an indolent growth pattern, yet malignant. Secondly, thymoma features strikingly different clinical characteristics among different histological types. What is more, thymoma is frequently associated with some autoimmune disorders, esp. myasthenia gravis^1–4^. Despite interesting pathological and biological characteristics, the tumourigenesis of thymoma remains largely to be determined. Like other malignant tumors, carcinogenesis of thymoma is also characterized by the stepwise accumulation of genetic and molecular abnormalities after carcinogen exposure. Most studies tried to address the tumorigenesis by characterizing genetic alteration on a single gene level^5–7^, and only a few systematic researches have been demonstrated^8,9^. Here, we reported our microarray analysis on gene expression pattern on thymoma samples, which demonstrated interesting features in thymoma, esp. myasthenia gravis. Our work may shed light on understanding of the genetic mechanism of tumorigenesis of thymoma and provide important molecular clues for different pathological types of thymoma.

## Results

From 2014 to 2016, we collected 31 pathologically confirmed thymoma. 5 cases were type AB, 6 B1-type cases, 12 B2-type cases, 5 B2B3-type cases, 3 B3-type. Among these samples, 6 cases were myasthenia gravis negative and 25 cases with myasthenia gravis. We then analyzed the transcriptome of these samples through microArray. Striking differences in gene expression were identified between thymoma and paratumoral tissues. 292 genes were upregulated more than 2-fold, 2 genes more than 5-fold. The Top 10 upregulated genes included: CCL25, HIST1H1B, SH2D1A, DNTT, PASK, CENPF, HIST1H2BD, S100A14, NPTX1; We also found that 596 genes were downregulated more than 2-fold, 115 genes more than 5-fold, 21 genes more than 10-fold, 6 genes more than 20-fold. The top 10 downregulated genes included: PLIN1, MYOC, ADH1A, FABP4, ADIPOQ, ADH1C, MGST1, LPL, CIDEC.

Among the genes upregulated for more than 2-fold, 6 genes were previously reported as driver oncogenes: FANCI, NCAPD3, NCAPG, OXCT1, EPHA1 and MCM2.

**Figure 1:**
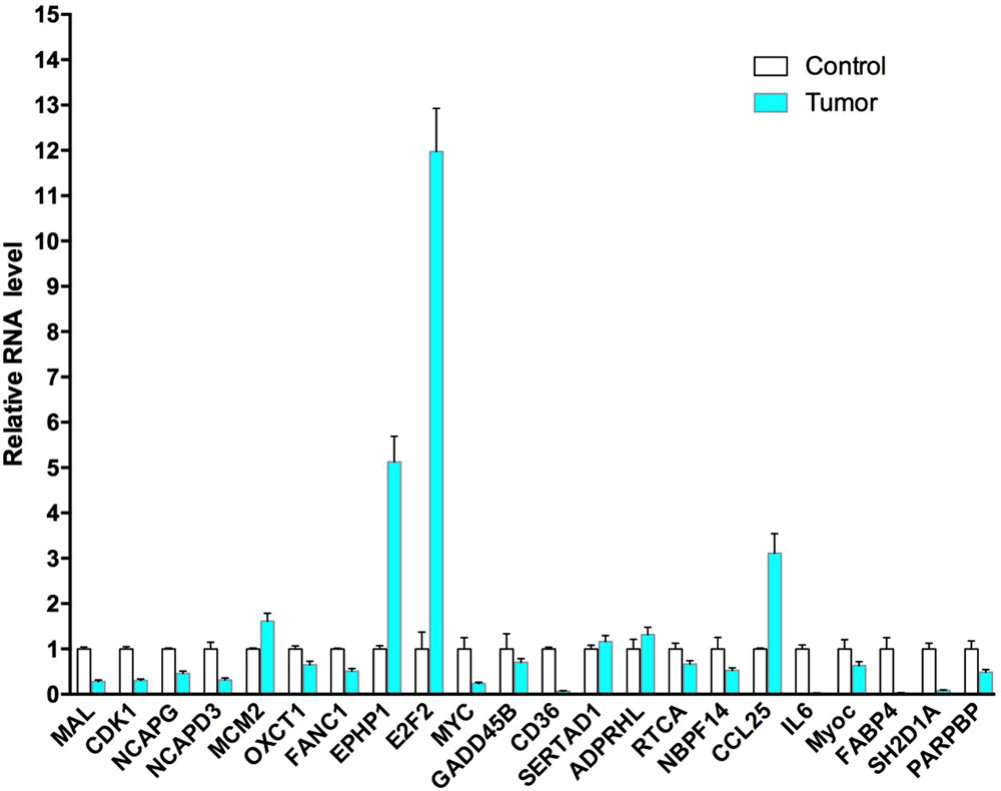
Among differentially expressed genes further confirmed by quantitative RTPCR, E2F2, EPHA1, CCL25 and MCM2 were upregulated significantly.

Up- and down-regulation of gene expression of these genes were validated by quantitative real-time PCR (RT-PCR, Fig. 1). We noticed 8 most dysregulated expression genes: E2F2, EPHA1, CCL25 and MCM2 were upregulated, while MYOC, FABP4, IL6 and CD36 were downregulated. In our series, EPHA1 increased significantly in 71.0% cases, and MCM2 increased significantly in 61.3% cases.

### Chromosome analysis of thymoma

**Figure 2:**
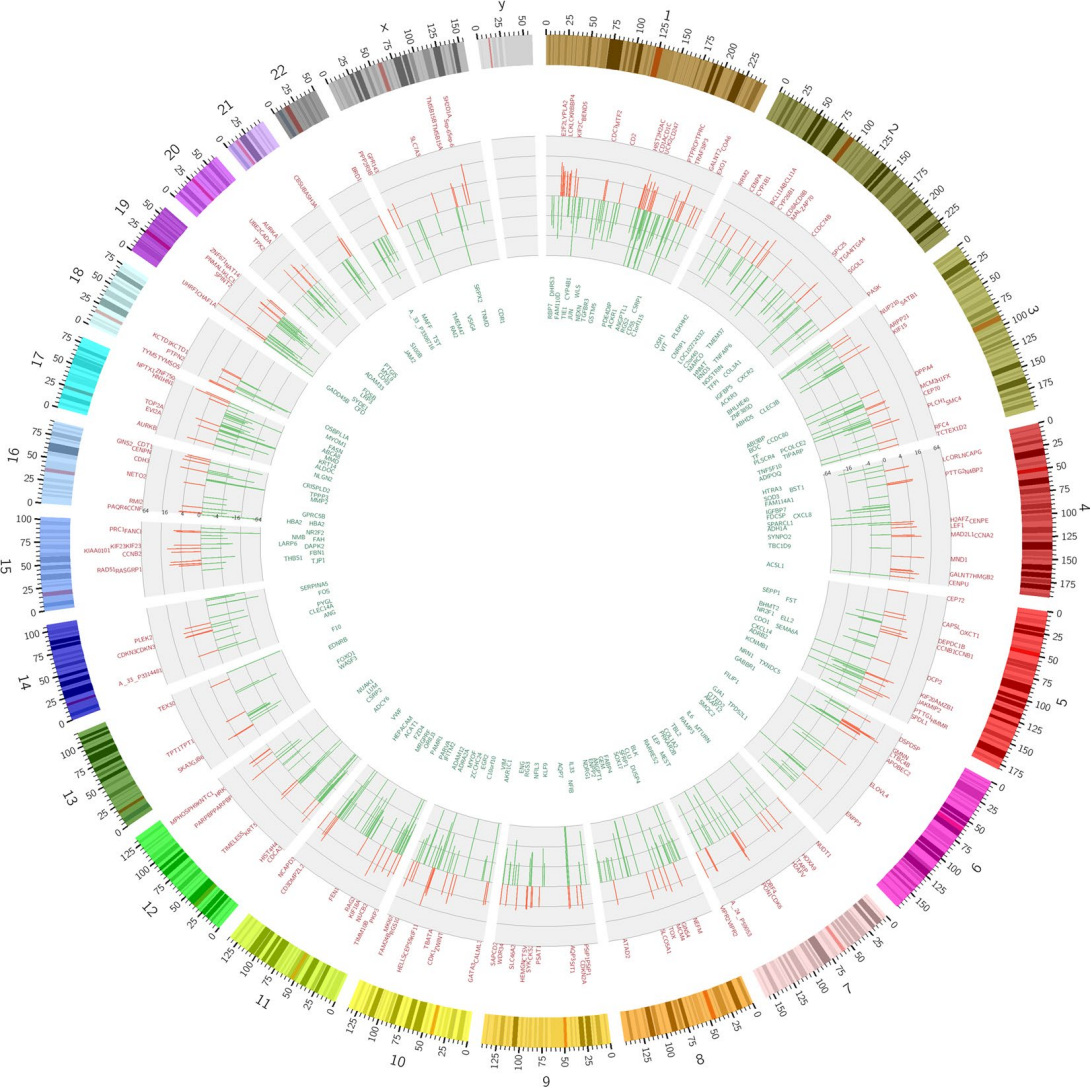
Chromosome analysis of thymoma shows that Genetic aberrations occurred mostly in chromosome 1 and 19. In chromosome Y, there were no genetic aberrations found.

Genes significantly upregulated mainly appeared in chromosomes 1, 2, 6, 10, 15, 19, while chromosomes 1, 3, 5, 7, 8, 10, 11, 12, 17, 19, had significantly downregulated genes. Genetic aberrations occurred mostly in chromosomes 1 and 19. Interestingly, chromosome Y seems to be clear of chromosomal aberrations (Fig. 2).

#### Unsupervised/supervised cluster analysis

**Figure 3:**
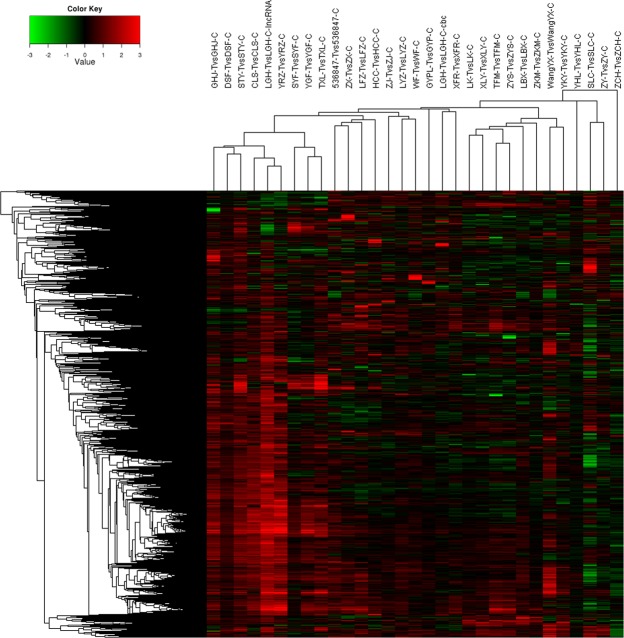
Unsupervised cluster analysis showed four distinct clusters, composed of 6, 3, 18 and 4 tumors, respectively.

**Figure 4:**
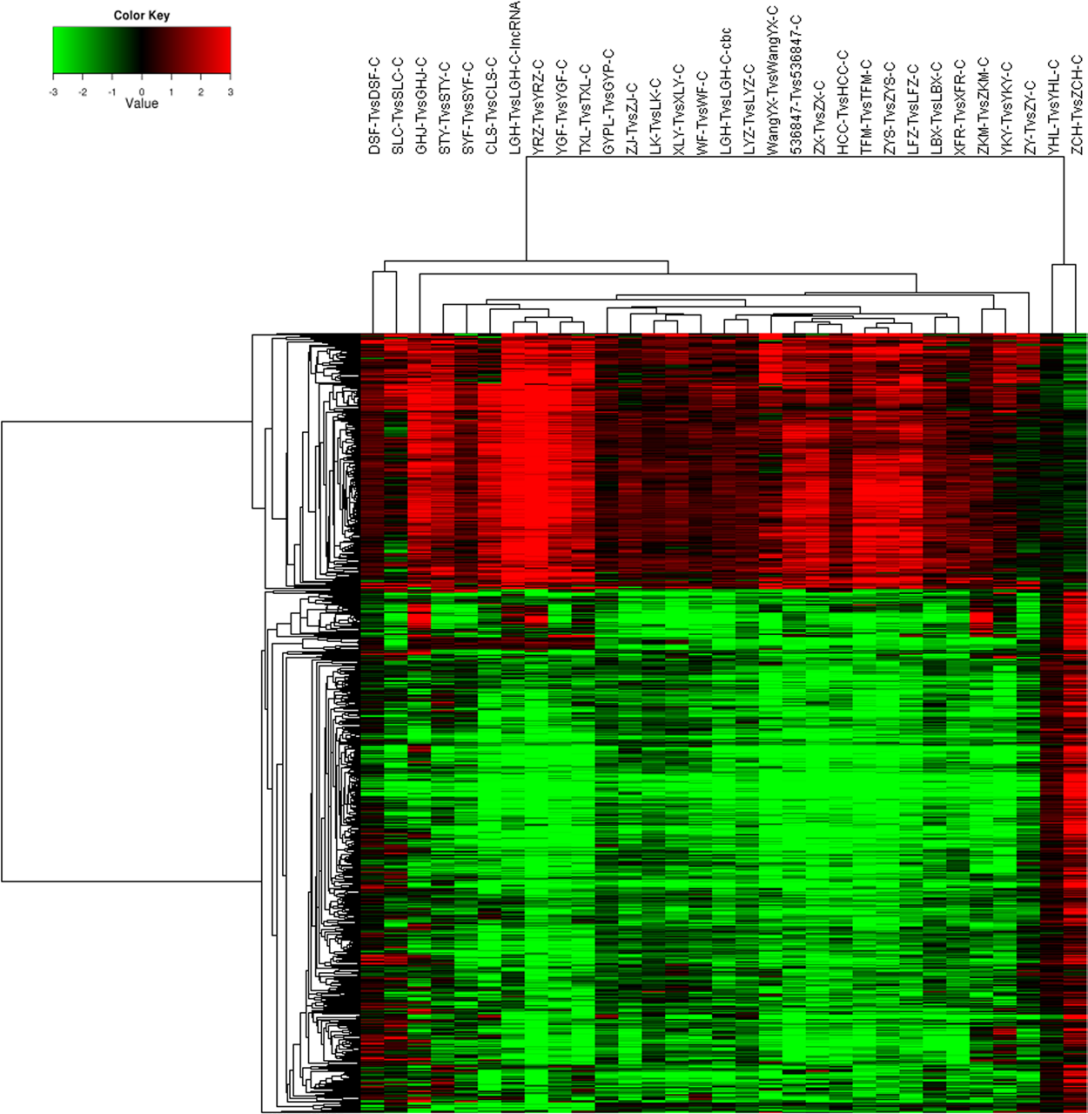
Supervised clustering heat map: 6 distinct clusters were identified.

Using 10000 genes, unsupervised cluster analysis showed four distinct clusters, comprised of 6, 3, 18 and 4 tumors, respectively. There was no cluster just comprised of thymoma of any particular histologic subtype (Fig. 3). We selected 2-fold upregulated and 2-fold downregulated genes to generate a supervised clustering heat map. 6 distinct clusters, composed of 2, 8, 7, 7, 4 and 3 tumors, respectively, were identified (Fig. 4). In cluster 1, two were type B2 tumors; in cluster 6, three were type B2/B3 tumors; Type B2 thymoma was also found in 3 out of the other four clusters, which did not have any specific histologic subtype.

#### Signaling pathways analysis

**Figure 5:**
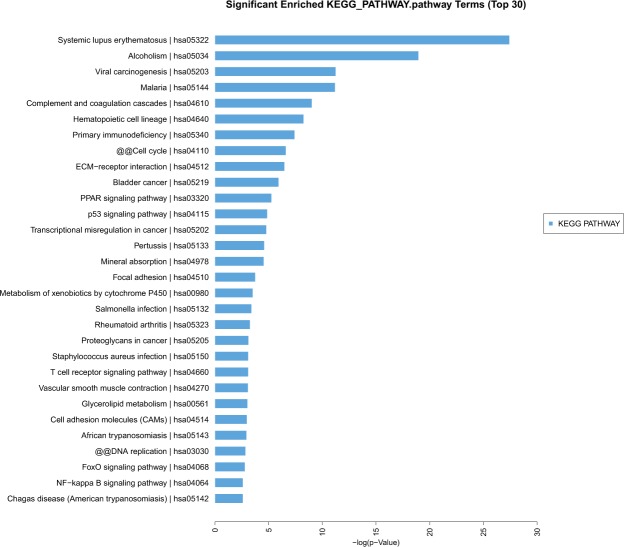
KEGG database analysis found several top signaling pathways maybe associated with the pathological process of thymoma, including Systemic lupus erythematosus, Viral carcinogenesis, Complement and coagulation cascades, Primary immunodeficiency, Cell cycle, ECM-receptor interaction, PPAR signaling pathway, p53 signaling pathway, Focal adhesion and so on.

KEGG analysis showed several signaling pathways were associated with thymoma at the molecular and cellular levels, which may provide important information for revealing the most significant biological functions of thymoma. The top ten signaling pathways included: Systemic lupus erythematosus; Alcoholism; Viral carcinogenesis; Complement and coagulation cascades; Hematopoietic cell lineage; Primary immunodeficiency; Cell cycle; ECM-receptor interaction; Bladder cancer; PPAR signaling pathway; p53 signaling pathway; Transcriptional misregulation in cancer; Pertussis; Mineral absorption (Fig. 5).

#### Comparison Between thymoma with and without MG

**Figure 6:**
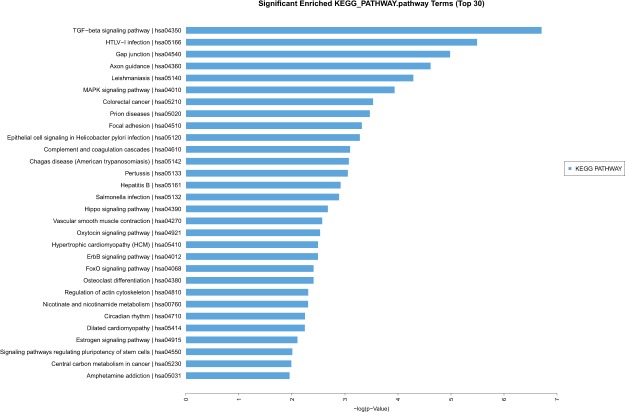
During comparisons between thymoma with and without MG, 2 signaling pathways with more than 2-fold upregulated genes (TGF- beta signaling pathway and HTLV-I signaling pathway)were found.

Thymoma are frequently associated with MG. However, the molecular mechanism remains to be determined. By comparing transcriptome of thymoma with and without myasthenia gravis, we found that 5 genes (PNISR, CCL25, NBPF14, PIK3IP1 and RTCA) were upregulated more than 2-fold, and that more than 30 genes were downregulated more than 2-fold. CCL25, NBPF14, PIK3IP1 were most upregulated; and GADD45B, SERTAD1, TNFSF12, MYC, ADPRHL1 were most downregulated. The expression levels of these 8 genes were verified through real-time PCR. Our KEGG analysis suggested that TGF-beta signaling pathway and HTLV-I signaling pathway were identified in genes upregulated for more than 2-fold in thymoma with myasthenia gravis in comparison to those MG negative samples, which suggested that these pathways may be responsible for MG in thymoma patients (Fig. 6).

## Disscusion

Thymomas are histologically heterogeneous tumors of thymic epithelial cells origins. Thymomas have a relatively good outcome among all forms of malignant tumors with a 5-year survival rate of over 70 percent and a 10-year survival rate of over 50 percent^10,11^. Like other malignant tumors, there are so many biological factors that contribute to the thymoma’s growth and proliferation^5–7^. But the exact molecular basis underlying tumourigenesis of thymoma still remains elusive.

Earlier researches show that changes in certain genes appear to be involved in thymic tumorigenesis^8,12–14^. Our data demonstrate that some gene mutations might play an important role in the pathogenesis of thymomas. These dysregulated genes were frequently expressed in chromosomes 1, 2, 3, 5, 6, 7, 8, 10, 11, 12, 15, 17, 19. Unlike other studies^8,12,14^, genes, such as EPHA1, CCL25, E2F2, MCM2, MYOC, FABP4, IL6 and CD36, were differentially expressed in our series. Our microarray results were highly intriguing. EPHA1 and MCM2 were regarded as driver genes formerly reported in cancer genomics. The expression levels of EPHA1 vary considerably in different types of normal tissues and tumors, or even in different phases of tumor development, suggestive of its functional pluralism^15,16^. In our study, the overexpression of EPHA1 was found in 71.0% cases. MCM2, one member of MCM family, expresses little in stationary phase while highly in proliferative and transformational phase. MCM2 accurately reflects the cell proliferation activity and is considered as a specific marker for carcinoma and precancerous lesions^17,18^. The overexpression of MCM2 is closely correlated with the genesis and development of tumors. In our study, the overexpression of MCM2 was noticed in 61.3% cases. Further studies of carcinogenic ability of EPHA1 and MCM2 may provide a way for the diagnosis and potential targets for treatment. CCL25, a small cytokine belonging to the chemokine family, is known as Thymus-Expressed Chemokine. CCL25 is believed to play a role in the development of T-cells. The gene for CCL25 is located on human chromosome 19^19^. Significant increased expression of CCL25 was noticed in 80% (20/25) cases of thymoma with MG, while only one case of thymoma without MG that had overexpression of CCL25, which suggested that overexpression of CCL25 gene in thymoma might play a vital role in the pathogenesis of myasthenia gravis. The E2F family plays a crucial role in the control of cell cycle and action of tumor suppressor proteins and is also a target of the transforming proteins of small DNA tumor viruses. Among differentially expressed genes further confirmed by quantitative RT-PCR, E2F2 located on human chromosome 1 is the most overexpressed gene in our study.

By KEGG database analysis, several important signaling pathways were identified. They were Systemic lupus erythematosus, Viral carcinogenesis, Complement and coagulation cascades, Primary immunodeficiency, Cell cycle, ECM-receptor interaction, PPAR signaling pathway, p53 signaling pathway, and Transcriptional misregulation in cancer. All of these pathways alterations were consistent with the malignant fact of thymoma^20,21^. Detailed functional study of these pathways may provide clues to understand the molecular bases of special features of thymoma. By comparisons between thymoma with and without MG, the HTLV-I signaling pathway was identified in genes upregulated for more than 2-fold in MG positive samples. Our result is in consistance with earlier reports that either HTLV-I or part of the virus genome was involved in the etiopathogenesis of myasthenia gravis^22–24^.

Similar to other studies^8,9^, we could not categorize thymoma by the unsupervised/supervised cluster analysis. The unsupervised/supervised cluster analysis did not correlate well with the histologic WHO classification. Certain histologic types of thymoma might exist in different clusters, while most clusters did not have any specific histologic subtype.

In conclusion, our study provided important information on the genetic mechanism of thymoma. It shed light on the molecular bases for analyzing the functional process of thymoma and finding potential biomarkers. It may also be helpful in understanding possible causes of MG in thymoma patients.

## Methods

From 2014 to 2016, we analyzed 31 thymoma (including 5 cases of type AB, 6 B1-type cases, 12 B2-type cases, 5 B2B3-type cases, 3 B3-type cases of thymoma; only 6 cases of thymoma were not associated with myasthenia gravis, 25 cases with myasthenia gravis) using CapitalBio mRNA microarray. All cases are primary tumors. Patients’ characteristics are summarized in Table 1. Apart from myasthenia gravis, these patients did not present other paraneoplastic disorders. Thymoma samples were collected during surgical procedures just from Beijing Tongren Hospital. The collected specimens were immediately frozen in liquid nitrogen. And then, they were kept at −80 °C refrigerator.

**Table 1:** Patients’ characteristics.

	patients
Number	31
Median age (range, years)	60 (27–74)
Male (n)	17
Female (n)	14
WHO histological type	
A B	5
B_1_	6
B_2_	12
B_2_/B_3_	5
B_3_	3
Masaoka’s clinical staging	
I	2
II	18
III	9
IV	2
Metastasis (n of cases)	Plural dissemination (2)

### RNA extraction, labeling and hybridization

Total RNA containing small RNA was extracted from thymoma and paraneoplastic thymic tissue by using the Trizol reagent (Invitrogen) and purified with mirVana miRNA Isolation Kit (Ambion, Austin, TX, USA) according to manufacter’s protocol. The purity and concentration of RNA were determined from OD260/280 readings using spectrophotometer (NanoDrop ND-1000). RNA integrity was determined by capillary electrophoresis using the RNA 6000 Nano Lab-on-a-Chip kit and the Bioanalyzer 2100 (Agilent Technologies, Santa Clara, CA, USA). Only RNA extracts with RNA integrity number values >6 underwent in further analysis.

### Microarray imaging and data analysis

The lncRNA + mRNA array data were analyzed for data summarization, normalization and quality control by using the GeneSpring software V13.0 (Agilent). To select the differentially expressed genes, we used threshold values of ≥2 and ≤−2-fold change and a Benjamini-Hochberg corrected p vlaue of 0.05. The data was Log2 transformed and median centered by genes using the Adjust Data function of CLUSTER 3.0 software then further analyzed with hierarchical clustering with average linkage. Finally, we performed tree visualization by using Java Treeview (Stanford University School of Medicine, Stanford, CA, USA).

#### RNA isolation, cDNA synthesis and Quantitative PCR (QPCR)

Tissue samples were homogenized by power homogenizer in 1 ml of TRIZOL reagent (Invitrogen). Then total RNA from all samples was isolated according to the manufacturer’s instructions. Complementary DNA (cDNA) was synthesised from 1 ug total RNA using the MMLV Reverse Transcriptase cDNA kit (TAKARA) as the manufacturer’s instructions.

Primers for qPCR reactions were designed to span intron boundaries and synthesized by Tsingke Biological Technology. All primer sequences for qPCR are listed as Table 1. Quantitative PCR (qPCR) was performed in 96-well plate using SYBR Premix Ex Taq (TAKARA) on Bio-RadCFX96 real-time PCR detection system (Bio-Rad). GAPDH primers were used as an internal control. The comparative Ct (ΔΔCt) method was used for data normalisation.

### Statistic analysis

All the data were presented as mean values ± standard deviations. Differences between experimental groups were compared with unpaired two-tailed t test. Statistical analyses were performed with GraphPad Prism 5.0, p < 0.05 was deemed to be statistically significant.

### Ethical approval

Because all patients in this study signed consent forms and were enrolled, informed consent was obtained from all participants. The study was approved by the Human Research Ethics Board of Beijing Tongren Hospital, Capital Medical University, and all experiments were performed in accordance with relevant guidelines and regulations.

## References

[1] Shelly S, Agmon-Levin N, Altman A, Shoenfeld Y (2011). Thymoma and autoimmunity. Cell Mol Immunol..

[2] Takai S (2017). Thymoma with immunodeficiency/Good syndrome associated with myasthenia gravis. Rinsho Shinkeigaku..

[3] Thomas A, Rajan A, Berman A, Giaccone G (2015). Multiorgan autoimmune manifestations associated with thymoma. J Thorac Oncol..

[4] Chen J (2011). Thymoma with pure red cell aplasia and Good's syndrome. Ann Thorac Surg..

[5] Mokhtar M (2014). Methylation and expression profiles of MGMT gene in thymic epithelial tumors. Lung Cancer..

[6] Tarrini G, Ciabatti E, Pacini S, Galimberti S, Petrini I (2017). GTF2I Mutations Are Common in Thymic Epithelial Tumors But Not in Hematological Malignancies. Anticancer Res..

[7] Song Z, Yu X, Zhang Y (2016). Rare frequency of gene variation and survival analysis in thymic epithelial tumors. Onco Targets Ther..

[8] Girard N (2009). Comprehensive genomic analysis reveals clinically relevant molecular distinctions between thymic carcinomas and thymomas. Clin Cancer Res..

[9] Lee GY (2007). Genome-wide genetic aberrations of thymoma using cDNA microarray based comparative genomic hybridization. BMC Genomics..

[10] Margaritora S (2010). Thirty-Five–Year Follow-Up Analysis of Clinical and Pathologic Outcomes of Thymoma Surgery. Ann Thorac Surg.

[11] Yu L (2012). Different Characteristics of Thymomas With and Without Myasthenia Gravis. Ann Surg Oncol..

[12] Cimpean AM, Encica S, Raica M, Ribatti D (2011). SOX2 gene expression in normal human thymus and thymoma. Clin Exp Med..

[13] Collins B (2013). Ikaros promotes rearrangement of TCR α genes in an Ikaros null thymoma cell line. Eur J Immunol..

[14] Belani R (2014). ASXL1 and DNMT3A mutation in a cytogenetically normal B3 thymoma. Oncogenesis..

[15] Shi X (2017). A role of the SAM domain in EphA2 receptor activation. Sci Rep..

[16] Herath NI, Doecke J, Spanevello MD, Leggett BA, Boyd AW (2009). Epigenetic silencing of EphA1 expression in colorectal cancer is correlated with poor survival. Br J Cancer..

[17] Fortuna D, Boman B, O’Neill R, Palazzo J (2017). MCM2 expression in serrated polyps demonstrates aberrant cellular proliferation. Hum Pathol..

[18] Yousef EM (2017). MCM2: An alternative to Ki-67 for measuring breast cancer cell proliferation. Mod Pathol..

[19] Maeda S (2012). Molecular cloning and expression analysis of the canine chemokine receptor CCR9. Vet Immunol Immunopathol..

[20] Panarese A, D’Andrea V, Pironi D, Filippini A (2014). Thymectomy and systemic lupus erythematosus (SLE). Ann Ital Chir..

[21] Engel P, Francis D, Graem N (1998). Expression of bcl-2 in fetal thymus, thymomas and thymic carcinomas. Association with p53 expression and review of the literature. APMIS..

[22] Manca N (2002). Angelini. Detection of HTLV-I tax-rex and pol gene sequences of thymus gland in a large group of patients with myasthenia gravis. J Acquir Immune Defic Syndr..

[23] Kikuchi K (2002). A novel animal model of thymic tumour: development of epithelial thymoma in transgenic rats carrying human T lymphocyte virus type I pX gene. Int J Exp Pathol..

[24] Tsuji T (2005). Malignant transformation of thymoma in recipient rats by heterotopic thymus transplantation from HTLV-I transgenic rats. Lab Invest..

